# Defending access to medicines in regional trade agreements: lessons from the Regional Comprehensive Economic Partnership – a qualitative study of policy actors’ views

**DOI:** 10.1186/s12992-021-00721-4

**Published:** 2021-07-08

**Authors:** Belinda Townsend

**Affiliations:** Menzies Centre for Health Governance, School of Regulation and Global Governance, Australian National University, Canberra, Australia

**Keywords:** Trade agreement, Access to medicines, Regional comprehensive economic partnership, Intellectual property, RCEP, TRIPS, TRIPS-plus, Civil society, Advocacy, Public health

## Abstract

**Background:**

The Regional Economic Partnership Agreement (RCEP) is a mega regional trade agreement signed by fifteen countries on 15 November 2020 after 8 years of negotiation. Signatories include the ten members of the Association of South East Asian Nations (ASEAN) plus China, New Zealand, Japan, South Korea and Australia. India was a negotiating party until it withdrew from the negotiations in November 2019. The RCEP negotiations were initially framed as focused on the needs of low income countries. Public health concerns emerged however when draft negotiating chapters were leaked online, revealing pressures on countries to agree to intellectual property and investment measures that could exacerbate issues of access to medicines and seeds, and protecting regulatory space for public health. A concerted Asia Pacific civil society campaign emerged in response to these concerns, and in 2019, media and government reporting suggested that several of these measures had been taken off the table, which was subsequently confirmed in the release of the signed text in November 2020.

**Results:**

This paper examines civil society and health actors’ views of the conditions that successfully contributed to the removal of these measures in RCEP, with a focus on intellectual property and access to medicines. Drawing on twenty semi-structured qualitative interviews with civil society, government and legal and health experts from nine countries participating in the RCEP negotiations, the paper reports a matrix of ten conditions related to actor power, ideas, political context and specific health issues that appeared to support prioritisation of some public health concerns in the RCEP negotiations.

**Conclusions:**

Conditions identified included strong low and middle income country leadership; strong civil society mobilisation, increased technical capacity of civil society and low and middle income negotiators; supportive public health norms; processes that somewhat opened up the negotiations to hear public health views; the use of evidence; domestic support for health issues; and supportive international public health legislation. Lessons from the RCEP can inform prioritisation of public health in future trade agreement negotiations.

## Background

The inclusion of intellectual property (IP) protections in bilateral and regional trade agreements can negatively impact access to generic medicines (i.e. non-originator medicines) through the extension of pharmaceutical monopolies [1–3]. This public health issue has received significant global attention leading to affirmations in the World Trade Organization’s (WTO) Doha Declaration on TRIPS and Public Health that countries have the right to interpret global IP rules ‘to protect public health and, in particular, to promote access to medicines for all’ [4]. The stalling of multilateral trade agreement negotiations at the WTO since Doha, however, has led to an increase in bilateral and regional trade agreements between smaller groups of countries, many of which have contained elevated levels of IP protection beyond the WTO Agreement on Trade-Related Aspects of Intellectual Rights (TRIPS), referred to as ‘TRIPS Plus’ measures [3, 5–7]. As of January 2021, for example, the United States had bilateral or regional trade agreements in force with twenty countries, all of which contained intellectual property chapters ([8], see also [9]).

TRIPS Plus IP measures included in recent bilateral and regional trade agreements include those that broaden the scope of patentability, such as for new methods of using known products; enable patent term extensions beyond the WTO mandated 20 year patent term; introduce monopolies on clinical trial data; link patent status with the marketing approval of generics; provide for trade secrets protection; and impose more stringent IP enforcement measures [10]. There is a growing body of literature analysing the negative impact of these provisions on access to medicines through delays to the timely entry of generic medicines [11–14].^1^ For example, a study of the impact of IP measures introduced in Jordan after signing the US-Jordan FTA determined delays to the market entry of generic medicines estimated to cost 18 million US dollars in 2004 alone [1].

These analyses and associated public health campaigning have led to several high-level multilateral Commissions as well as joint government declarations promoting the full use of TRIPS flexibilities [15] and explicit opposition to the inclusion of TRIPS Plus measures in trade treaties [16, 17]. The United Nations’ Secretary General’s High Level Panel on Access to Medicines, for example, argues that the expansion of IP in bilateral and regional agreements beyond TRIPS is inconsistent with public health objectives, and that government ‘failure to conduct robust impact assessments before such agreements are signed is tantamount to a neglect of state duties to safeguard the right to health’ [18]. Public health scholars and policy-makers continue to lament, however, that public health and access to medicines remain largely on the periphery of trade policymaking, with little government use of health impact assessments or other regulatory tools to prioritise health [19].

In this context, greater understanding of the governance conditions which enable countries to resist TRIPS Plus measures in trade negotiations would contribute to an emerging knowledge base on what works and why for elevating public health concerns in trade agreements [20–25]. In the field of international relations, scholars have pointed to constraining and enabling conditions such as asymmetries in economic power between negotiating countries [26], the particular configuration of negotiation blocs and their negotiating strategies [27], and domestic interests and constituents, including the role of civil society and their framing strategies [28]. In the field of public health, key governance challenges identified include issues of transparency, accountability, participation, integrity and capacity [20], with public health actors operating within a dominant neoliberal paradigm that privileges corporate interests [29, 30]. A small set of empirical studies in public health focused on what has worked points to conditions such as requirements for consultation with health experts and public health actors; transparency around trade negotiations; and mandated use of health impact assessments [29–34]. A recent analysis of Australia’s participation in the CP-TPP agreement, for example, found that domestic concerns for access to medicines and protecting regulatory space for tobacco control emerged onto the government’s agenda through a combination of conditions involving actor networking, framing, supportive policy processes, favourable evidence and public support [34].

This paper contributes to this emerging knowledge base through an analysis of policy actors’ views of the conditions that enabled prioritisation of access to medicines norms in the Regional Comprehensive Economic Partnership Agreement (RCEP) - a mega regional trade agreement recently signed by 15 countries in the Asia Pacific region. It discusses results from key informant interviews with public health and public interest-oriented civil society actors and government health officials intimately involved in lobbying and monitoring the RCEP negotiations, from nine participating countries. The RCEP agreement is explained below, along with the theoretical framework, before the Results explore the key conditions emerging from the interviews, and implications are discussed.

### The Regional Comprehensive Economic Partnership agreement

In 2012, the ten member countries of the Association of South-East Asian Nations (ASEAN) began negotiations for a mega regional trade agreement with six countries they had existing bilateral agreements with, namely Australia, New Zealand, Japan, South Korea, China and India. The agreed Guiding Principles document released that year suggested that the negotiations would be focused on the development needs of the low and middle-income (LMIC) ASEAN country members [35]. For some, RCEP was seen as a competing agreement to the then US-led Trans Pacific Partnership (TPP) under negotiation at the time with several of the RCEP countries, but which notably excluded China and India [36]. For others, RCEP and the then TPP were seen as ‘building blocks’ towards an eventual mega regional trade agreement for the Asia Pacific [37].

RCEP became controversial for access to medicines advocates in 2015 when leaked IP chapters by Japan and South Korea revealed that these two countries were seeking the inclusion of several TRIPS Plus IP standards in the agreement [38, 39]. These included provisions to broaden the scope of patentability, allow for patent term extensions, patent linkage, data exclusivity, and the seizure of IP-infringing goods in-transit [40]. Analysis showed that, if agreed to, several of the LMIC would have had to amend their domestic IP laws, with serious concerns for negative impacts on timely access to generics [40]. Access to medicines advocates in the region and transnational health advocates began to focus on the RCEP through public health campaigns [41, 42]. In June 2019, indications in the media and in statements by those monitoring the negotiations suggested that many of these TRIPS Plus measures were no longer on the negotiating table [43]. This was confirmed in the release of the final text of the agreement which was made available after RCEP was signed on 14 November 2020 by the fifteen parties (not including India who withdrew in late 2019). The final text showed that RCEP contained no pharmaceutical specific intellectual property provisions, including measures to extend patent terms, the scope of patents, data exclusivity, or patent linkage [44].

The RCEP agreement also did not contain requirements for countries to accede to the International Convention for the Protection of New Varieties of Plants (UPOV 1991) nor did it include an investor-state dispute settlement mechanism (ISDS), both of which had become controversial for public health during the trade discussions [45]. UPOV 1991 was heavily criticised by civil society and public health actors because several of the LMIC had not acceded to the Convention due to the potential impacts of introducing stringent patent rules on access to seeds for small scale farmers and thus for food security [40]. The potential for RCEP to include ISDS, a measure which would enable foreign companies to make claims of compensation against RCEP countries if they infringed investor rights, was also controversial during the negotiations, including for its potential constraining impact on space for public health regulation [45].

This paper reports on the views of civil society and government health officials who monitored and lobbied governments throughout the RCEP negotiations on what conditions they perceived as appearing to enable greater prioritisation of public health concerns in the RCEP agreement. The analysis focuses on conditions which supported the removal of TRIPS Plus measures on pharmaceuticals, however several informants also reflected on dynamics they saw as shaping the opposition to patents on seeds in UPOV 1991 and on including ISDS in the agreement.

### Theoretical framework

Shiffman and Smith’s framework of political prioritisation guided the study [46]. This framework posits that global health issues are more likely to receive political prioritisation when there is a convergence of interest, ideational, institutional and issue-based conditions. First, the power of actors involved including the strength of individuals and organisations and their cohesiveness is highlighted as an important condition. Second, ideas and framings used by actors to oppose or support an issue play an important role. Third, the political context including policy processes and structures can shape the uptake of issues. Finally, the particular characteristics of health issues themselves, such as the strength of evidence, is important. This framework was used to guide the study protocol questions and analysis.

## Methods

Twenty interviews were conducted with key policy actors from public health and public interest civil society organisations, experts and government officials active in monitoring and advocacy on the RCEP agreement in the Asia Pacific region. Eighteen participants were principally located in nine of the participating RCEP countries (14 in LMIC, 4 in high income), and two were from transnational NGOs. Interviewees were identified using purposive and snowballing sampling based of the authors’ knowledge of key organisations in the region and on referral from interviewees.

Interviews were conducted between July 2019 and March 2020, averaged 45–60 min duration, and were conducted in English. Informants were asked about their views of the barriers and enablers to advancing attention to health concerns in the RCEP negotiations. Prior to interviews, the author developed a semi-structured protocol of questions based on the theoretical framework including categories of actors, ideas, political context and issue characteristics (See Table 1). Consent was obtained from participants and interviews were recorded, except one participant who requested that notes be taken instead of audio recording.

**Table 1:** Framework concepts and example questions

Key Concept	Example interview questions
Actors	• Who were the main actors seeking to influence the RCEP negotiations? Who was most influential? Why?
Ideas	• How were actors framing their interests? Were any frames supportive of public health issues?
Political context	• What institutional processes, either formal or informal and inside or outside the trade negotiations did you see as important? Why? • Were there turning points or events that shifted the agenda over this time period (this could include changes in government, shifts in public opinion, government reports, and external events)?
Issue characteristics	• Were there reasons specific to health issues that helped or hindered prioritisation? What role did evidence play? Or domestic or international legislation?

Interview recordings were transcribed verbatim and coded using NVivo 11 qualitative data management software. The initial coding scheme was informed by the theoretical framework and final codes developed through an iterative process involving inductive analysis of the data with reference to theoretical concepts from the literature. Analysing informant accounts thematically allowed for identification of core factors and conditions which shaped the advancement of access to medicines concerns in the RCEP negotiations. This coding was based on the majority of informants’ views. Interview findings were triangulated with supplementary data including policy actors’ submissions on their organisational websites, government website reports and media reporting of the negotiations. Ethics approval was received from The Australian National University Human Research Ethics Committee (Protocol 2019/326).

## Results

Interviews indicated several interest, ideational, institutional and issue-based conditions that appeared to enable greater attention to the issues of access to medicines, access to seeds, and protecting regulatory space for public health in the RCEP negotiations. The matrix of conditions identified are classified in the categories of actors, ideas, political context and issue characteristics in Table 2 and detailed below.

**Table 2:** Conditions identified by informants as supporting attention to access to medicines, seeds and protecting public health regulatory space in the RCEP negotiations

Framework Concepts	Conditions
Actors	• Strong leadership by low and middle income ASEAN members • Strong leadership by India • Civil society mobilisation domestically and regionally • Technical expertise role of civil society advisers and experts with negotiators
Ideas	• Strong access to medicines norm established
Political Context	• Lessons learnt from the Trans-Pacific Partnership negotiations • ‘Breaking open’ the negotiations to alternative views
Issue characteristics	• Strength of evidence • Domestic salience of health issue • Existing international legislation for health issue

### Actors

1. Strong leadership by low and middle income ASEAN members

The leadership of key middle-income countries in ASEAN, particularly Malaysia, Philippines and Indonesia, was highlighted by several informants as an important condition for influencing ASEAN’s negotiating position on IP and ISDS. The ten ASEAN member states negotiated RCEP as a bloc and were oriented to a consensus-based negotiating position. The vocal influence of these countries against TRIPS Plus and ISDS was highlighted as important for setting the ASEAN position:

“ASEAN speak as one, and when you have Singapore as Chair of ASEAN, it’s very difficult for Singapore to say what it might apply to farmers or patients in Myanmar or Laos, where countries have a high number of people living in rural areas. People who also don’t have good access to healthcare…. I thought Indonesia and the Philippines played a balancing role in ASEAN in focusing on those impacts.” (NGO #14).

“Indonesia is one where I would say was at the forefront in terms of what really trying to steer, and push the whole coalition to continue to stick to TRIPS and not moving beyond that” (NGO #13).

“ASEAN countries were critical of the ISDS provisions and were pushing… to not have this mechanism under RCEP” (NGO #15)

The role of these middle-income countries in steering RCEP away from TRIPS Plus was also highlighted by several informants as important in the context of least developed countries (LDC) negotiating capacities (i.e. Cambodia, Laos and Myanmar). Informants noted that initial leaked draft RCEP IP text indicated proposals for LDCs to sign on to TRIPS Plus provisions as soon as they graduated from LDC status, yet LDC negotiating officials they spoke to seemed unaware of the implications. Instead, they relied on other middle income countries to resist IP measures beyond TRIPS:

“LDCs were not even aware that there was a discussion in the RCEP IP chapter which could have meant they were signing on to TRIPS Plus provisions…there was a complete lack of expertise” (NGO #13)

“it was very clear that the LDC had no capacity to evaluate the text… [on the leaked document on LDC extension] we couldn’t find a single LDC champion to take up that issue” (NGO #9).

2. Strong leadership by India

India was also identified as a particularly influential actor on the outcome of the negotiations for IP and ISDS. India participated in the RCEP negotiations until it withdrew in November 2019. By that time, media and government reports indicated that the pharmaceutical TRIPS Plus IP measures and ISDS were off the table [47, 48]. Several informants reported that the Indian negotiators were more willing to speak with public health NGOs and advocates on the issues of access to medicines and to raise these issues in the RCEP public forums and media surrounding the negotiations:

“the Indian negotiators and the interest around India and medicines and the fact that India plays such an important role in global health was to their advantage and what I felt helped remove the IP provisions” (NGO #9)

India’s ultimate withdrawal from RCEP in late 2019 was the result of several factors. A government note cited issues around trade deficits with the other RCEP countries, a lack of market access assurances, and issues around tariff reductions, while Prime Minister Modi also cited negative effects for farmers, small and medium enterprises and the dairy sector in a public speech announcing India’s withdrawal [49]. Informants also noted a groundswell of domestic opposition in India against RCEP that had mounted both from business and a coalition of civil society organisations:

“the whole Indian business was against it, the national security establishment was against it, civil society was against it, farmers were against it… within Cabinet, three or four Ministries opposed it” (NGO #17)

“Patients came out very strongly, along with the farmers groups, pretty much thousands of people. I think everyone from outside India was a bit surprised to see the number of people who gathered to protest RCEP. It was a very strong voice” (NGO #14).

3. Civil society mobilisation domestically and regionally

A third key factor was the strong mobilisation of civil society groups domestically and regionally. Informants noted the formation of a regional RCEP network of civil society organisations and researchers focused on a range of public interest issues in the negotiations, including access to medicines, access to seeds and protecting space for public regulation. This network formed through an email list serve and met face-to-face on the sidelines of several RCEP negotiating rounds and online:

“when civil society came together in Kuala Lumpur to agree to look at RCEP and build a regional campaign, in each country we started to see coordination taking form” (NGO #14).

“RCEP connected people in not all 16 but I think probably 18 countries at least, and they’ve all been very active in sharing information… and they have managed to get information from their countries and share extensively on many issues…. It was very useful sharing experiences and also being able to decide on how to move forward strategically” (NGO #13)

“whenever there was a round of negotiations, CSO organisations worked to coordinate a meeting with other CSOs from other RCEP countries… it’s like an opportunity to get together and lobby negotiators (NGO #17).

Many informants viewed the informal network as a place to try and overcome the lack of transparency around the negotiations – to share information, ideas on public health campaigning strategy, and how to work both inside and outside the negotiations to drive the public health messaging:

“if we got information from government, through informal or formal ways, we would check with the other NGOs in other countries in the network” (NGO #8)

“we had an insider and outsider strategy from the beginning. Some focused on going to negotiations, doing details, monitoring the negotiations, trying to influence the text… other concentrated on public education, mobilising public support, having rallies” (NGO #11).

The role of civil society was also highlighted by some as pivotal in driving attention to the issue of access to seeds in the context over debates over UPOV 1991 rules on patents on seeds, which were ultimately not included as a requirement in the final text:^2^

“What happened was the UPOV 1991 [rules on IP for seeds] stayed in for a very long time… so we started to talk to other civil society.. I remember talking to Thai colleagues who had built a strong movement of farmers. And they pushed back on that side. And ASEAN only started to move on UPOV 91 when civil society in those ASEAN countries started to fight back” (NGO #9)

4. Technical expertise role of civil society advisers and experts with negotiators

Finally, the technical role of civil society and government health experts to both LMIC country negotiators and to other civil society groups was highlighted as influencing the resistance to TRIPS Plus in RCEP, particularly for providing alternative language for countries to consider. Related to this expertise was the establishment of working relationships between these expert advisors and government trade negotiators:

“With the Director General of the Trade Negotiation Minister, we know each other. We have face to face and private communications sometimes. Like if the Director General has made a comment publicly that is not in favour of health… we can have communication and say ‘hmm the statement you made is not really good’. We communicate together” (Government #16).

“We have a good working relationship with other civil society organisations. So they support us in terms of suggesting the issue that we sometimes do not realise there is an issue. And they also use the information from us to advocate… we work together” (NGO #13).

“We have a good relationship with the head negotiator, because we keep talking and build trust, we share our information and analysis to them… so it depends on building up trust” (NGO #8).

### Ideas

1. Access to medicines norm

In terms of ideas, a key enabling factor highlighted by many informants was a pre-existing ‘access to medicines’ norm in public discourse which facilitated resistance to TRIP Plus measures. Many informants spoke of access to medicines within the language of human rights– of the right to health and access medicine as a human right. Informants also spoke of access to medicines as a life necessity, with lack of access harmful and potentially deadly. This focus on access to medicines was front and centre in campaign and advocacy around RCEP and IP. ‘Access to medicines’ was a frame that appeared to be internalised by advocates and some government negotiators and thus a dominant norm in discussions about trade, IP and health. Informants noted that many government trade negotiators they spoke to articulated this norm in conversation. For example:

“[trade] negotiators would raise the issue about health and access to medicines. … Some would say ‘I have family who suffer from HIV’, who said it was a personal issue for them as well as for patients, for patients with chronic diseases. So it seemed like many of them knew about this issue and it was something they agreed with” (NGO #14).

In contrast, the arguments for extending IP for medicines were framed as creating monopolies for private companies, which did not hold the same moral discursive influence as the right to health and affordable medicines. Nonetheless, only some areas of IP were successfully framed as issues for access to medicines in the RCEP negotiations. The issue of TRIPS Plus IP enforcement, for example, was one issue that advocates struggled to get onto the mainstream agenda, both within the civil society network and in conversations with government trade negotiators. The final text of RCEP includes TRIPS Plus measures on IP enforcement that could create barriers for access to medicines. Advocates reported the issue of IP enforcement as more difficult to frame within the language of access and human rights, and also identified gaps in evidence needed to help support this framing.

### Political context

1. Lessons learnt from the Trans Pacific Partnership Agreement

The dynamics and lessons from the Trans Pacific Partnership agreement (TPP) negotiations, much of which was conducted in parallel with RCEP, was also seen as a key factor shaping the outcome for TRIPS Plus in RCEP and for lobbying around ISDS. The then US-led mega regional TPP was negotiated by sixteen countries in the Pacific Rim before the US withdrew under President Trump in 2017. Remaining parties, many of which were also negotiating the separate RCEP agreement, renegotiated the TPP as the renamed Comprehensive Partnership on Trans-Pacific Partnership (CP-TPP) signed 8 March 2018. Informants identified the formation of an informal coalition of access to medicines and public health actors in response to the TPP from 2010 onwards as an important precursor to the RCEP by which many organisations became familiar with the issues of IP and access to medicines and trade treaties:

“the coalition was already in place, and not starting from scratch thanks to the TPP, and we had been following… there was already a big group following trade negotiations [on access to medicines] and many countries were helpful in sharing their concerns and keeping pressure up” (NGO #13).

A key outcome was the suspension of many controversial TRIPS Plus IP measures in the CP-TPP in 2018 after the US withdrew from the agreement [50]. This was a particularly important dynamic highlighted by several informants as enabling resistance to TRIPS Plus in the RCEP:

“It was definitely helped by the fact that they [IP measures on medicines] were suspended in the CP-TPP… including countries that wanted them excluded like New Zealand and Australia too” (NGO #94)

“The withdrawal of the US government and suspension of the IP provisions in the CP-TPP had an impact on RCEP” (NGO #8).

Informants also identified lessons from the TPP negotiations on ISDS as informing their approach in RCEP. Most notably, many informants saw the tobacco ‘carve-out’ in the CP-TPP, which allows member countries to elect to deny ISDS claims applying to tobacco control measures [51], as problematic for broader public health and public interest concerns regarding ISDS. Drawing on this lesson, many informants spoke of pushing a broader anti-ISDS position in RCEP rather than a single-issue carve-out. Informants also reflected that there was not an active anti-tobacco lobby group around RCEP like there had been in the TPP, which likely also influenced the outcome away from a carve-out to no agreement on ISDS:

“I was sceptical of carving out tobacco… it doesn’t help us on other health issues… we didn’t feel we were doing a good job at investment, by doing piecemeal kind of safeguards. They would just put a Band-Aid on one and then another would spout… so we learned a lot. And our position is now very clear – ISDS has to go” (NGO #9)

Finally, many informants saw the years of political contestation around the TPP/CPTPP as shaping a desire by RCEP negotiators to ‘get a deal done’ and not get stuck on controversial issues:

“actually the issue [of IP and medicines] was really hard for them to achieve an agreement… so that is why, they dropped it… so they conclude the agreement as soon as possible after that” (NGO #15).

“in the context of the US and China, the RCEP countries are eager to signal they are willing to recognise the importance of cooperation, and they wanted to show it is possible to get an agreement at the regional level” (NGO #6).

2. ‘Breaking open’ the negotiations to alternative views

Informants spoke of a concerted effort by several public interest and public health actors to ‘break open’ the RCEP negotiations to enable public health views to be heard. This pressure appeared somewhat successful with the RCEP negotiating rounds shifting from an initial closed space, where civil society actors were not invited, to several later rounds allowing some form of participation through civil society presentations and informal engagement with negotiators:

“With the RCEP there was nothing for the first couple of years. There was no opportunity for civil society at all to meet with the negotiators at negotiation times, at each round. But civil society did attempt to make contact with negotiators… but there wasn’t the same formal setup [as there was in TPP initially] at each negotiating round” (NGO #11).

“So for civil society it was like homework for us, to track down where it [negotiating round’] might happen. And then we pushed for a space, for a hearing. Sometimes they provided an hour or hour and a half for civil society to speak” (NGO #14).

Several informants saw this shift as important for airing public health concerns. They encountered barriers, however, when negotiating rounds were held in ASEAN countries that did not support civil society, and many saw the contrasting TPP processes as being more open for engagement than the RCEP:

“When it took place in countries that had strong civil society movements, there was often some amount of opportunities for us to access the negotiation, to varying degrees of course. But it was noticeable when the negotiation happened in China, Vietnam or Laos, we would be like ‘ok, we can’t do anything’… but in Thailand or Australia, New Zealand, there was more opportunity for us to do something, both inside and outside the negotiations” (NGO #20).

“In the TPP we actually forced quite extensive opportunities for conveying the research and views of communities, including professional health communities, to negotiators. And that was very influential early on in the negotiations… it introduced negotiators to people who knew what they were talking about so relationships were built over time. It also gave governments who were themselves reticent to stuff they could point to… so the democracy and secrecy issues were right from the start… and it was easier to do it in TPP than RCEP because RCEP had ASEAN and China.” (Expert # 94)

Overall, informants remained ultimately dissatisfied with the level of engagement with RCEP negotiators and contrasted the limited opportunities for public health and civil society engagement with formal RCEP industry forums that had run from the beginning of the negotiations:

“a senior employee of a tech firm I knew emailed me asking some advice on ecommerce, and he forwarded me emails with draft RCEP negotiation texts attached from the government… it meant the government was showing the text to industry and asking for their advice” (NGO #92)

“Industry were given two days consultations for the business sector, and only one and a half hours for civil society. That’s the different space and time we were given for consulting” (NGO #14).

As part of ‘breaking open’ the space for engagement, informants also reflected on the importance of accessing leaked negotiating texts in order to inform their analysis of potential health concerns in RCEP:

“Having leaks is very important to understand, especially in countries where English is not the main language… having that in advance is very important for civil society to put pressure on government. Otherwise we don’t have any basis if we don’t see what has been discussed” (NGO #14).

“After that [the leaks] they realised that they had to talk to us, if only to explain their version of what was going on” (NGO #11).

Some informants also reflected on engagement with parliamentarians outside the RCEP negotiations in their countries as part of the strategy to push for opening a space for engagement with negotiators:

“At first they [negotiators] refused to meet us, so I pressed the National Assembly and they contacted government officers and said ‘why didn’t you meet with civil society? Why didn’t you listen to them?” (NGO #92).

### Issue characteristics

1. Strength of evidence

The availability of clear evidence on the causal links between IP measures and access to medicines was seen as particularly useful for resisting TRIPS Plus proposals on pharmaceuticals in RCEP:

“We have a lot of information and research regarding the TRIPS Plus issue and impact on public health, particularly for access to medicines” (NGO #8).

“Officials from so many of the RCEP countries were shocked, like ‘what, a thousand dollars?” [on cost of medicines] and how patents were acting as barriers and how there are TRIPS flexibilities that should be used. So those kind of practical examples were useful for negotiators to understand” (NGO #13).

In addition, personal stories were also seen as useful for highlighting the potential impacts of TRIPS Plus measures on access to medicines, and on UPOV 1991 rules on patents on seeds:

“Farmers feel the impact of corporate control of seeds. So it’s not difficult then to link it to how RCEP with the UPOV provision would further create an environmental in corporate control.. and farmers mobilised against it effectively” (NGO #6).

“The moment you add patient voices to it, that is like clear cut evidence, and it has an impact… the moment you put a human face to it” (NGO #13).

However, evidence was seen as an insufficient condition on its own to influence the negotiations. As one informant lamented:

“oh evidence isn’t enough. It is necessary but not sufficient…. It’s important to show credibility of critique in the public domain and with negotiators... it’s important to get the health community onside… it’s important if you’re having a public debate, including dealing with hostile media and providing evidence… but it’s certainly not sufficient. There has to be pressure at multiple levels, whether it is just the core public health actors saying “hands off our health”, through to effective lobby groups like trade unions and non-health entities like trade unions, environmental activities and so on” (Expert #94).

2. Domestic salience of health issue

The domestic salience of health issues was also seen as an important issue-specific condition that helped resistance to TRIPS Plus, UPOV 1991 and ISDS in RCEP. Several informants noted the ongoing issues of the price of medicines, rising healthcare costs, and ongoing concerns regarding food security and support for farmers in many of the LMIC RCEP countries as important domestic factors. Several countries, both high and low income, had also experienced ISDS arbitration and high profile ISDS cases. The release of India’s draft Bilateral Investment Treaty [52] in September 2015, which proposed an alternative investment model to ISDS, was also seen as an influential development for the discussions on ISDS in the RCEP negotiations:

“you had Indonesia with its very extensive health insurance program, you had sensitivities in Malaysia on HIV/AIDS and you also had Vietnam’s sensitivity on that… so there was a level of awareness even before public health advocates focused on this in RCEP… and this had provided more leverage for those arguments to the point where many of the controversial elements are off the able because of the collective concern” (Expert #94).

“Australia, New Zealand and India started to feel the heat on ISDS… the Australians were upset with an Indian company Adani threatening to sue them over mining… and on the other hand India wanted out of ISDS because were constantly threatened with lawsuits… so I think it was something that many countries were sensitive about” (NGO #9).

3. Presence of existing international legislation

Finally, informants highlighted the presence of international law and legislation, particularly the WTO 2001 Doha Declaration on TRIPS and Public Health [4] as a supportive condition for resisting TRIPS Plus.

“We now have a level of government awareness from Doha, even before you have the expert input from public health advocates and so on bringing the evidence to support the positions that the governments want to take. So that has provided more leverage for those arguments to the point where some of the most controversial elements are now off the table” (Expert #94).

## Recommendations for reforming the treaty-making process

While the RCEP negotiations indicated successful resistance to TRIPS Plus measures on pharmaceuticals, it is important to note that the final text of the agreement did go beyond TRIPS on matters of IP enforcement [44]. This expanded regime for IP enforcement remains an issue for public health yet has received little attention in public and media discourse and in wider civil society campaigning. Furthermore, while the final RCEP text does not include an ISDS mechanism, it does include a commitment by the parties to enter into discussions on settling disputes between parties and investors within 2 years of the agreement entering into force – meaning discussions on ISDS (and public health concerns regarding ISDS) will likely re-emerge. Analysis of the final text has also indicated other concerns for health that remain including, but not limited to: rules on trade in goods that discourage government assistance for local industry, an issue highlighted by the COVID-19 pandemic; rules that facilitate foreign investment in services liberalisation and restrict the ability of government to regulate; and no commitments on labour rights or environmental standards [53]. Furthermore, a recent impact analysis of tariff liberalisation under RCEP has shown that ASEAN’s trade balance will worsen by 6% per annum, a loss of a significant source of income for public health expenditure in several ASEAN countries [54].

All informants noted problems of transparency and lack of engagement with negotiators as limiting understanding of the wider potential health impacts of trade treaty negotiations:

“In our country there is no formal mechanism for civil society to submit their views, and no transparency or openness to information. … we have to proceed with sending letters and asking for consultation. So it’s only started from our efforts to have consultation. But there is no formal mechanism we can access” (NGO #15).

Furthermore, informants noted a lack of engagement between government trade and health officials in participating countries as exacerbating a lack of attention to health concerns:

“When we tried to bring up this issue [of access to medicines] with the Ministry of Health they said that they are not being involved in the discussion” (NGO #12).

“We see very little participation by the Department of Health, and very little capacity when we engaged them, they knew very little about what’s happening with the trade negotiations” (NGO #6).

In the context of a lack of transparency and public health and civil society engagement, informants identified potential reforms to better aid prioritisation of health issues in future trade negotiations. These included; the need for independent government bodies to conduct Health and Human Rights Assessments; formal civil society forums at the national and trade agreement negotiation level; release of draft texts of country positions during the negotiations and before an agreement is signed; and the involvement of parliamentary bodies in scrutinizing these texts and canvassing experts, including public health experts, on potential health impacts.

Thailand’s previous 2007 Constitutional rules that required health impact assessments, transparency of trade frameworks of negotiation, reports to Parliament, and Parliamentary approval before Thailand signed trade agreements was highlighted by some informants as an example of success, although the removal of these provisions under the recent coup d’état was seen as a step backwards. Nevertheless, Thailand’s ongoing government International Health Policy Programme was highlighted as a key program for providing health impact assessments of trade agreements in Thailand. Indonesia’s recent legal decision to require Parliament to debate trade agreements before they are signed was also highlighted as a positive example, yet informants noted that the law has yet to be implemented.

Finally, informants reflected on the need for greater civil society engagement and mobilisation on trade agreements in the Asia Pacific region. This included the need for building technical expertise and capacity building and national and transnational civil society networking:

“we need to bring more civil society on board… so there are a handful of networks or organisations who are always there, and the success is pretty small but we want to mobilise on a huge scale so we have a bigger impact… and a problem is resources, which are really stretched for us” (NGO #17).

## Discussion

This study builds on existing literature examining the governance conditions that shape greater attention to health in trade agreement negotiations [3, 20–26, 34, 55–58]. Specifically, the analysis identifies ten conditions reported to have enabled resistance to controversial TRIPS Plus IP measures on pharmaceuticals, rules requiring parties accede to UPOV 1991 rules on patents on seeds, and ISDS in the RCEP negotiations. This section reflects on these findings for prioritising public health in future trade agreement negotiations.

First, the formation of an informal regional civil society network of public interest and public health actors from the participating RCEP and surrounding countries was highlighted by several informants as bolstering the public health campaign in the region, drawing media and government attention to several public health concerns. This network supported NGO and health actors to overcome some of the barriers around lack of transparency and formal rules for engagement with trade officials at both the national and regional level. The network enabled exchange of information on what issues were reportedly under discussion in the trade negotiations, what evidence could be collected and provided to negotiators and governments, and what messaging was needed for public debate. The formation of this informal network appeared to serve a critical function in supporting community groups and public health groups in many of the participating countries.

This network also appeared to play a crucial role in ‘breaking open’ the RCEP negotiations from an initial closed space to a semi-invited space that enabled public health experts and NGOs to raise public health issues with trade negotiators. While the various RCEP stakeholder-negotiating rounds varied in their level of access to civil society and public health experts, the opening up to NGOs and public health actors enabled informants to meet with trade negotiators and raise public health evidence and analysis through formal and informal avenues. This use of formal and informal policy processes has been documented as an important strategy for public health in trade negotiations elsewhere [24, 34].

Within this network, technical experts also played an important role in providing evidence and analysis to civil society groups and to government negotiators. This technical advisory role highlights the importance of trade and health literacy for governments, NGOs and wider civil society [59]. The finding suggests that trade and health literacy programs for public health NGOs and health officials could be strengthened in the Asia Pacific region through, for example, WHO regional and country offices, in partnership with academic and technical NGO experts.

There is also significant scope for a broadening of the public health and public interest movements that monitor the public health impacts and issues in trade agreement negotiations in the region, particularly to include the wider social determinants of health beyond medicines (e.g. noncommunicable diseases, environment, climate, labour, gender) [60, 61]. The RCEP informal network involved several trade unions, trade justice, access to medicines, and some gender justice organisations from countries in the region, but there appeared to be less engagement from public health and noncommunicable disease advocacy organisations and climate justice groups.

Second, the strong leadership against TRIPS Plus measures on pharmaceuticals by key ASEAN member states such as Malaysia and Indonesia was also crucial for informing ASEAN’s position against TRIPS Plus measures on pharmaceuticals. This was bolstered by India’s outspoken position against TRIPS Plus. While the mix of high and low-income countries in each regional trade negotiation varies, as do their offensive and defensive interests for public health, the findings re-affirm the importance of LMIC countries negotiating as a bloc and holding firm lines on public health issues [27]. It is worth noting that most of the RCEP countries were IP-importing nations, and so the absence of offensive interests on IP for pharmaceuticals was also likely a key condition which shaped resistance to TRIPS Plus.

Third, the role of evidence was highlighted as important although insufficient on its own to influence trade negotiations. In the study, informants saw the issues of medicine pricing, healthcare costs and costs of ISDS cases in many of the RCEP countries as shaping governments positions on these issues in RCEP. Informants particularly utilised causal evidence generated over the past decade on the retrospective impact of IP measures on affordable access to generic medicine supply [1], and prospective analysis of the potential impact of these IP measures [6, 10–14]. Personal stories were also used to drive home the equity impacts that IP on seeds and medicines could have for communities in many RCEP countries. Scholarly analysis of the potential impacts of leaked IP proposed measures in several ASEAN countries and India [40] was also presented to government trade negotiators at the RCEP stakeholder rounds. Most studies appeared to be prospective analyses, which is understandable given the time lag for many LMIC in introducing TRIPS [9]. It does demonstrate however a need for more retrospective analysis to quantify the impacts of TRIPS Plus in countries which have introduced these and similar measures. The role of evidence suggests fertile ground for further research on analysing the impacts of trade on social, commercial and environmental determinants of health.

Related to evidence was the supporting role of international legislation and global norms on public health, particularly access to medicines. The WTO Doha Declaration on TRIPS and Public Health [4] was identified by informants as an important global instrument invoked when countering TRIPS Plus proposals in the negotiations. International debates on ISDS at the time of RCEP negotiations, such as the EU’s decision to terminate its internal ISDS arrangements and not agree to ISDS in future trade agreements with other nations [62] was another exogenous development shaping global debates. The findings suggest that there could be greater promotion of international norms and legislations in other public health areas for trade officials, such as the UN Sustainable Development Goals [63], UN Human Rights Conventions and WHO legislation and norms on health issues such as global alcohol control [64].

Finally, the findings reaffirm the need for structural reforms in trade policymaking processes both at the national level and in how regional trade agreement negotiations are conducted [20, 24, 29, 34]. As highlighted above, informants identified power asymmetries between trade and health officials within governments, as well as a lack of transparency and formal processes for engagement with public health experts and NGOs and wider civil society. These power asymmetries have been documented elsewhere, including at the global level [23]. All informants were highly critical of forms of engagement introduced at the national level in RCEP countries, mostly perceived to be superficial and one-sided. Informant’s views of the power of industry actors in the negotiations, and their significant access to negotiators, reaffirms other analyses of the power that industry and transnational corporate actors wield in trade policy [3, 22, 26, 29, 65, 66].

Informants identified a range of measures needed in their respective countries and in regional negotiations to provide better prioritisation to health issues, including independent government bodies to conduct Health and Human Rights Assessments; formal civil society forums; release of draft texts during the negotiations and before an agreement is signed; and the involvement of parliamentary bodies in scrutinizing these texts and canvassing experts, including public health experts.

### Limitations and further research

This study principally focused on the views of public health and public interest actors active in monitoring the RCEP negotiations. As such, it did not include the views of government trade negotiators. Trade officials’ views have been documented in other countries in other studies [34]. This is the first study in a larger project of research that will canvass trade actor and politician views on trade and health in the Asia Pacific region.

These findings may also have applicability beyond the trade domain for thinking about mechanisms for prioritising health in government policy. Further health research could apply this framework of conditions to cases in the social and commercial determinants of health to identify the range of conditions that enable or constrain health, and thus reveal potential strategies that generate successful prioritisation of health in economic sectors. Further application of this framework of other trade agreements and in other countries could generate additional valuable lessons on specific health topics and specific country context.

## Conclusions

Initial proposals for the inclusion of TRIPS Plus IP measures on pharmaceuticals, requirements to accede to UPOV 1991 rules on patents on seeds, and ISDS in the RCEP agreement negotiations raised significant public health concerns. Informants identified ten conditions that appeared to contribute to greater attention to the issues of access to medicines, access to seeds, and protecting regulatory space for public health in the RCEP negotiations. These findings aid our understanding of the factors that can enable or constrain attention to health in trade agreement negotiations, and offer insights for potential pathways to elevate greater attention to health in regional trade negotiations in the future.

## Data Availability

The data that support the findings of this study are available on request from the corresponding author BT. Interview data are not publicly available due to them containing information that could compromise research participant privacy.

## Footnotes

1 The impact of TRIPS Plus varies according to a country’s existing IP commitments and domestic law when they sign a trade agreement. Many LMIC began introducing TRIPS IP requirements later through using TRIPS flexibilities, meaning the impacts of TRIPS Plus measures are still prospective (see [9]).

2 Instead, the RCEP IP chapter includes language that parties may seek to cooperate with other parties if they intend to ratify a range of multilateral agreements, including UPOV 1991 (Article 11.9 3(a)).
